# Synchronous Diagnosis of Multiple Myeloma, Breast Cancer, and Monoclonal B-Cell Lymphocytosis on Initial Presentation

**DOI:** 10.1155/2016/7953745

**Published:** 2016-05-10

**Authors:** A. Vennepureddy, V. Motilal Nehru, Y. Liu, F. Mohammad, J. P. Atallah

**Affiliations:** ^1^Department of Internal Medicine, Staten Island University Hospital, 475 Seaview Avenue, Staten Island, NY 10305, USA; ^2^Department of Pathology, Staten Island University Hospital, 475 Seaview Avenue, Staten Island, NY 10305, USA; ^3^Division of Hematology and Oncology, Staten Island University Hospital, 475 Seaview Avenue, Staten Island, NY 10305, USA

## Abstract

The cooccurrence of more than one oncologic illness in a patient can present a diagnostic challenge. Here we report an unusual case of concomitant existence of multiple myeloma, breast cancer, and monoclonal B-cell lymphocytosis on initial presentation. The challenge was to accurately diagnose each disease and stage in order to maximize the therapeutic regimen to achieve cure/remission. Successful management of the patient and increased life expectancy can be achieved by multidisciplinary management and patient-oriented approach in multiple primary malignant synchronous tumors.

## 1. Introduction

Multiple primary malignant tumors (MPMTs) are rarely observed in clinical practice; however, certain fundamental factors for the potential etiology have been described in the literature, including the environment and behavior (tobacco, occupation, pollution, and ultraviolet light), genetic predisposition (Li-Fraumeni or Beckwith-Wiedemann syndromes), previous medical treatment (radiotherapy or chemotherapy), and complex interaction between all these factors [1]. The association between varying cancer types can be classified into two categories, which are dependent on the timing of their discovery. An American review stated that the tumors are synchronous when the cancers occur at the same time or within 2 months of each other, whereas metachronous tumors occur when the cancers follow in sequence more than 2 months apart [2]. The majority of MPMTs that occur in multiple organs are metachronous, while the presence of synchronous lesions is less common, and in accordance with the behavior of malignancy lesions, these tumors are more frequent with aging.

This distinction implies important diagnostic and therapeutic challenges. From a diagnostic point of view the different patterns of MPMs should be considered. Therapeutically, a multidisciplinary and patient-oriented approach should be considered.

## 2. Case Report

A 77-year-old female with history of hypertension, diabetes mellitus type II, dyslipidemia, and chronic kidney disease stage II presented to our hospital after a fall initially suspected to be secondary to mechanical factors without any loss of consciousness. Her lab tests on admission were found to be as follows: hemoglobin of 10.8 g/dL (baseline was 12.5 g/dL) with red blood cell indices within normal limits, white blood cell count of 9.8 × 10^9^ cells/L with absolute lymphocyte count of 1.4 × 10^9^ cells/L, absolute granulocyte count of 7.8 × 10^9^ cells/L, and normal platelets. Her serum creatinine was 2.01 (baseline was 1.03) with a GFR of 29 and normal serum electrolytes. The patient's initial trauma workup and physical exam revealed a palpable mass in the upper outer quadrant of the left breast and multiple lytic lesions on C2 and C5 vertebral bodies and T2 spinous process on the computerized tomography (CT) scan. Subsequent magnetic resonance imaging (MRI) noncontrast of the brain and cervical spine exhibited multiple focal lesions of the calvarium, clivus, C2 and C5 vertebral bodies, and T1 spinous process and cord compression of C3-C4, C4-C5, C5-C6, and C6-C7 suggestive of metastatic disease. The patient was started on dexamethasone while undergoing malignancy workup. She then underwent a nuclear medicine bone scan of the whole body which showed multiple bony abnormalities in the left femoral shaft, left acetabulum, L1, L2, and multiple bilateral ribs consistent with metastatic disease.

**Figure 1:**
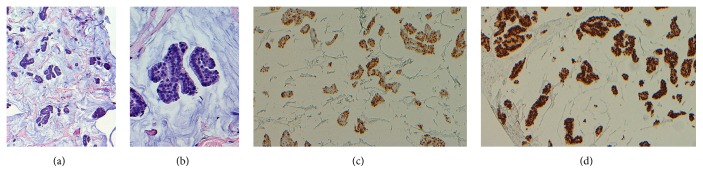
Colloid carcinoma of the breast. This invasive ducal carcinoma exhibits dominant mucin production and tumor cells are arranged in clusters and nests, floating in pools of extracellular mucin (a). Tumor cells are uniform in size and shape with hyperchromatic nuclei without prominent nucleoli and mitosis (b). PR (c) and ER (d) are positive in tumor cells. [(a) H&E, ×100; (b) H&E, ×400; (c) IHC, ×100; (d) IHC, ×100.]

The patient's mammogram and left breast ultrasound showed a 7.5 × 3.7 × 7.8 cm heterogeneous irregularly shaped mass at the 2:00 position, 15 cm from the nipple on the upper outer quadrant of the left breast. Subsequent biopsy of the mass revealed an invasive well differentiated ductal carcinoma with mucinous features (colloid carcinoma) (Figure 1). The breast cancer proved to be triple positive for estrogen, progesterone receptors, and HER2 via immunohistochemistry and fluorescence in situ hybridization testing. The lytic lesions on bone scan were initially suspected to be of metastatic breast cancer.

**Figure 2:**
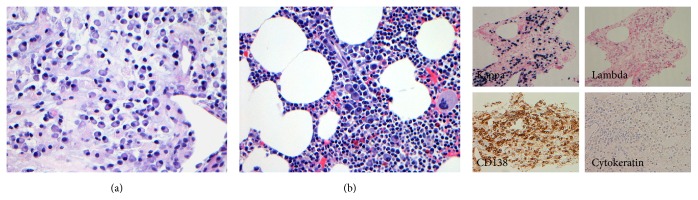
Photomicrographs from femur bone biopsy (a) and bone marrow clot preparation (b) showing a monomorphic population of plasma cells intermixed with few small lymphocytes and other hematopoietic cells in the bone marrow. The plasma cells show mature cytological features with strong CD138 reactivity and restriction for kappa light chain. No morphologic evidence of carcinoma is identified in both specimens and staining with cytokeratin marker is negative. [(a) H&E, ×100; (b) H&E, ×400.]

The combination of acute kidney injury, normocytic normochromic anemia, and multiple bony lesions was suspected of being multiple myeloma (MM) and its workup was done by serum and urine electrophoresis, serum and urine immunofixation, and free light chain ratio. The results revealed abnormal IgA kappa monoclonal protein with elevated free light chain ratio. The patient's subsequent bone marrow biopsy confirmed the diagnosis of MM but also revealed a minute monoclonal B-cell population with typical chronic lymphocytic leukemia (CLL)/small lymphocytic lymphoma (SLL) phenotype confirmed via flow cytometry (Figure 2).

The patient improved significantly with the supportive treatment and was discharged from the hospital with outpatient follow-up. The patient was then started on REVLIMID and Decadron for MM and pertuzumab, trastuzumab, and letrozole for invasive ductal carcinoma of the breast. She underwent positron emission tomography (PET-CT) as part of the initial treatment workup as well as for determining the appropriate site for bone biopsy to differentiate between metastatic breast cancer and MM as MM lesions usually do not light up on the bone scan. The PET-CT revealed a fluorodeoxyglucose (FDG) avid 4.3 × 5.1 cm partially calcified left breast mass (SUV 3.2) as well as increased uptake (SUV 4.3) in the 5.3 × 4.3 cm lytic lesion of the right distal femur. The right femur and left pubic bone were biopsied which showed only monomorphic population of plasmacytoid cells consistent with plasma cell neoplasm with no evidence of metastatic breast cancer (Figure 2).

The patient is currently continuing the neoadjuvant chemotherapy with pertuzumab, trastuzumab, and letrozole while awaiting surgical evaluation for the triple positive localized left breast cancer and REVLIMID and Decadron for symptomatic multiple myeloma. She is also being monitored for the progression of the CLL/SLL clone.

Synchronous diagnosis of two different primaries is very important as the management of these coexisting malignancies is different. In our case report, we initially thought the lytic lesions could be from metastatic breast cancer which in turn proved to be pure myelomatous involvement of bone and no signs of metastases from breast cancer which changed the management and made the patient undergo mastectomy. This patient should also be monitored for any evidence of development of CLL as we found monoclonal B-cell population with typical CLL/SLL phenotypic picture on flow cytometry of bone marrow biopsy.

## 3. Discussion

Primary soft tissue extramedullary plasmacytoma is uncommon and is defined as a malignant tumor of plasma cells arising in the soft tissue in the absence of bone involvement. It can occur in any organ as a solitary form of plasma cell neoplasm [3]. Breast plasmacytoma is one of the major differential diagnoses in synchronous diagnosis of breast cancer and multiple myeloma as plasmacytomas may mimic breast cancer in some cases, especially when axillary lymph nodes are involved. Few case reports have been published in the literature where breast plasmacytomas can occur either as a solitary lesion without evidence of systemic disease [4–7] or concurrently with multiple myeloma [8–11] or as a sign of relapse in patients with known history of myeloma [12–15].

To the best of our knowledge, few case reports have been described in the literature with synchronous diagnosis of multiple myeloma and breast cancer. Cao et al. reported a case of breast tumor where infiltrating ductal carcinoma of the breast and breast plasmacytoma occurred which was coexistent in the breast tumor on initial presentation and the patient got successfully treated with chemotherapy for breast cancer and radiotherapy for plasmacytoma [16]. Khalbuss et al. reported a case of synchronous diagnosis of multiple myeloma and breast cancer with plasmacytoid morphology on initial presentation [17]. This case illustrated the deceptive cytomorphologic similarities between an epithelial malignancy and a hematopoietic malignancy. Since myeloma plasma cells vary from mature forms to pleomorphic, anaplastic plasma cells, they can easily mimic the cytomorphology of adenocarcinoma and vice versa. The distinction may be challenging by cytomorphology alone [17]. Kherfani et al. reported a case of spinal cord compression from concurrent multiple myeloma and metastatic breast cancer in the same vertebra [18]. Al-Said Ali et al. reported synchronous diagnosis of multiple myeloma and breast cancer on initial presentation similar to our case in 2009 [19].

## 4. Conclusion

In summary, here we presented a case of synchronous diagnosis of MM, breast cancer, and monoclonal B-cell lymphocytosis on initial presentation. The diagnosis of dual malignancy in a patient at the time of the first presentation is rare. We initially suspected the lytic lesions on bone scan could be from metastatic breast cancer but were found out to be only localized breast cancer with pure myelomatous involvement of bony lesions. This made the patient undergo mastectomy along with continuation of neoadjuvant chemotherapy. The patient also needs to be monitored for any development of CLL from monoclonal B-cell lymphocytosis. Recognizing both malignancies at the time of presentation and before initiation of treatment is crucial since the management of these coexisting malignancies is different.

In conclusion, successful patient's management and increased life expectancy can be achieved by multidisciplinary management and patient-oriented approach in multiple primary malignant synchronous tumors.

## References

[1] Rabbani F., Grimaldi G., Russo P. (1998). Multiple primary malignancies in renal cell carcinoma. *Journal of Urology*.

[2] Howe H. L. A review of the definition for multiple primary cancers in the united states.

[3] Van Nieuwkoop C., Giard R. W. M., Veen H. F., Dees A. (2001). Extramedullary plasmacytoma of the breast simulating breast cancer. *Netherlands Journal of Medicine*.

[4] Cutler C. W. (1934). Plasma-cell tumor of the breast with metastases. *Annals of Surgery*.

[5] Innes J., Newall J. (1961). Myelomatosis. *The Lancet*.

[6] Proctor N. S. F., Rippey J. J., Shulman G., Cohen C. (1975). Extramedullary plasmacytoma of the breast. *Journal of Pathology*.

[7] Bloomberg T. J., Glees J. P., Williams J. E. (1980). Bilateral breast lumps: an unusual feature of extramedullary plasmacytoma. *British Journal of Radiology*.

[8] Rosenberg B., Attie J. N., Mandelbaum H. L. (1963). Breast tumor as the presenting sign of multiple myeloma. *The New England Journal of Medicine*.

[9] Maeda K., Abesamis C. M., Kuhn L. M., Hyun B. H. (1973). Multiple myeloma in childhood: report of a case with breast tumors as a presenting manifestation. *American Journal of Clinical Pathology*.

[10] Bassett W. B., Weiss R. B. (1979). Plasmacytomas of the breast: an unusual manifestation of multiple myeloma. *Southern Medical Journal*.

[11] Ben-Yehuda A., Steiner-Saltz D., Libson E., Polliack A. (1989). Plasmacytoma of the breast—unusual initial presentation of myeloma: report of two cases and review of the literature. *Blut*.

[12] Craft I. L. (1967). The late appearance of extramedullary lesions in myelomatosis. *British Journal of Cancer*.

[13] Mangalik A., Gupta P. K. (1974). Soft tissue involvement in plasmacytoma and multiple myeloma: a report of seven cases. *Indian Journal of Pathology and Microbiology*.

[14] Ross J. S., King T. M., Spector J. I., Zimbler H., Basile R. M. (1987). Plasmacytoma of the breast. An unusual case of recurrent myeloma. *Archives of Internal Medicine*.

[15] Lombardi C., Calvi A., Bonera E., Savio A., Savio E. (1992). Bilateral breast involvement in a 71-year-old white man with lambda light chain disease. Regression after a new chemotherapy combination. A case report. *Tumori*.

[16] Cao S., Kang H.-G., Liu Y.-X., Ren X.-B. (2009). Synchronous infiltrating ductal carcinoma and primary extramedullary plasmacytoma of the breast. *World Journal of Surgical Oncology*.

[17] Khalbuss W. E., Fischer G., Ahmad M., Villas B. (2006). Synchronous presentation of breast carcinoma with plasmacytoid cytomorphology and multiple myeloma. *Breast Journal*.

[18] Kherfani A., Amri K., Hachem M., Abid L., Bouaziz M., Mestiri M. (2014). An association of vertebral breast cancer metastasis and multiple myeloma, revealed by a spinal cord compression. *Pan African Medical Journal*.

[19] Al-Said Ali A., Al-Bader I., Al-Ali F., Elgazzar A. H., Fayez S. (2009). Breast cancer and multiple myeloma at initial presentation. *Breast Journal*.

